# The impact of professional and organizational identification on the relationship between hospital–physician exchange and customer-oriented behaviour of physicians

**DOI:** 10.1186/1478-4491-13-8

**Published:** 2015-02-17

**Authors:** Jeroen Trybou, Gaelle De Caluwé, Katrien Verleye, Paul Gemmel, Lieven Annemans

**Affiliations:** Department of Public Health, Ghent University, Ghent, Belgium; Department of Innovation, Entrepreneurship and Services Management, Ghent University, Ghent, Belgium; Faculty of Medicine and Health Sciences, Vrije Universiteit Brussel, Brussels, Belgium

**Keywords:** Physician, Hospital, Perceived organizational support, Distributive justice, Customer-oriented behaviour

## Abstract

**Background:**

Hospitals face increasingly competitive market conditions. In this challenging environment, hospitals have been struggling to build high-quality hospital–physician relationships. In the literature, two types of managerial strategies for optimizing relationships have been identified. The first focuses on optimizing the economic relationship; the second focuses on the noneconomic dimension and emphasizes the cooperative structure and collaborative nature of the hospital–physician relationship. We investigate potential spillover effects between the perceptions of physicians of organizational exchange and their customer-oriented behaviors.

**Methods:**

A cross-sectional study was conducted on 130 self-employed physicians practicing at six Belgian hospitals. Economic exchange was measured using the concept of distributive justice (DJ); noneconomic exchange was measured by the concept of perceived organizational support (POS). Our outcomes consist of three types of customer-oriented behaviours: internal influence (II), external representation (ER), and service delivery (SD).

**Results:**

Our results show a positive relationship between DJ and II (adjusted *R*^2^ = 0.038, *t* = 2.35; *p* = 0.028) and ER (adjusted *R*^2^ = 0.15, *t* = 4.59; *p* < 0.001) and a positive relationship between POS and II (adjusted *R*^2^ = 0.032, *t* = 2.26; *p* = 0.026) and ER (adjusted *R*^2^ = 0.22, *t* = 5.81; *p* < 0.001). No relationship was present between DJ (*p* = 0.54) or POS (*p* = 0.57) and SD. Organizational identification positively moderates the relationship between POS and ER (*p* = 0.045) and between DJ and ER (*p* = 0.056). The relationships between POS and II (*p* = 0.54) and between DJ and II (*p* = 0.99) were not moderated by OI. Professional identification did not moderate the studied relationships.

**Conclusion:**

Our results demonstrate that both perceptions of economic and noneconomic exchange are important to self-employed physicians’ customer-oriented behaviours. Fostering organizational identification could enhance this reciprocity dynamic.

## Introduction

In recent years, the importance of patient-centred care has expanded and has increasingly taken over the caregiver-oriented model. With its integration by the Institute of Medicine as one of the six domains of quality, patient-centred care has received new attention [1]. In this study, we turn our focus on the patient-oriented behaviour of physicians in hospitals. Physicians hold a centrally important function in hospitals and are critical to hospitals’ organizational success [2]. For many years, physicians and hospitals have worked together in providing health care to the communities they serve. Overall, hospitals provide the resources in which the care can be delivered and facilitate physicians in delivering medical care [3]. Arguably, for hospitals to be successful in delivering patient-centred care, they must rely on their medical staff that make clinical decisions and interact intensively with the patient [4].

Against this background, hospital executives are charged with the development of organizations in which patient-centred care is efficiently delivered in an increasingly competitive environment [5]. Moreover, hospitals face a more competitive health-care environment because of increased patient mobility [1] and higher levels of consumer behaviour [6].

Ever since the groundbreaking work of Hesket and colleagues [7], it has been clear that spillover effects exist between the perceptions of employees of an organization and the perceptions of customers. More precisely, it appears that HR practices of organizations not only affect employee attitudes such as job satisfaction and organizational commitment but also have a “spillover effect” onto customers (e.g. customer satisfaction and service quality perceptions). This effect refers to the dynamic in which employee attitudes seem to spill over onto customers in service encounters. Following this line of thought, improvements in the workplace environment of service employees might be expected to increase extra-role customer-oriented behaviour [8]. In the literature, two types of managerial strategies for optimizing hospital–physician relationships have been identified. The first focuses on optimizing the economic relationship; the second focuses on the noneconomic dimension and emphasizes the cooperative structure and collaborative nature of the relationship between hospital and physician [9]. In this paper, we study both approaches from a social exchange perspective. More precisely, we apply the concepts of distributive justice [10] and perceived organizational support [11] to study the exchange relationships that hold between physicians and hospitals. Distributive justice (DJ), which pertains to the economic dimension, refers to the perceived fairness of the outcomes or rewards that an individual receives from the organization [12]. Perceived organizational support (POS) can be described as the global beliefs concerning the extent to which the organization values the employees’ contributions and well-being [11].

While these two concepts have been used frequently to understand the generic employee–organization relationship, few studies have applied these concepts to the hospital–physician relationship. Our objective in this work is to fill this gap by investigating these concepts in a sample of self-employed physicians. We make three important contributions to the literature. First, despite the importance of patient-centred care [13] and the abundant literature on social exchange [14], a limited number of studies have concentrated on the relationship between these two [8]. Second, while most studies of hospital–physician relationships have focused solely on the financial ties [9], we study both the economic and the noneconomic sides of the hospital–physician exchange. Third, it has been shown that, in the case of professionals, reciprocity is more complex than originally conceptualized [15]. More precisely, we turn to social identification in order to study the effects of organizational and professional identification in this dynamic of reciprocity. This refers to the extent to which the physician identifies with the hospital and the medical profession. These feelings of belongingness or oneness are thought to have powerful effects on how individuals interpret and react to perceived exchange with the organization [16].

The objectives of the present study are (i) to examine the effect of physicians’ perceptions of economic and noneconomic exchange on their customer-oriented behaviours and (ii) to investigate the moderating effects of physicians’ professional and organizational identification.

## Theoretical background

### Customer-oriented behaviour

Over the past few decades, scholars have asserted that a spillover effect exists between organizational members’ attitudes—such as perceptions of justice and job satisfaction—and customers’ satisfaction and service quality perceptions [17]. Previous studies have generally supported this assertion across several service industries [7]. Customer-oriented boundary-spanning behaviour can be interpreted as extra-role behaviour directed at “customers” [18].

In the services management literature, three different types of customer behaviour that link the organization to its customers have been conceptualized [19]. Firstly, employees play an important part in representing the organization to outsiders. They shape the image of the organization and the legitimacy through their advocacy of the organization. External representation therefore focuses on the organizational member as a vocal advocate to outsiders of the organization’s image, goods, and services [19].

Secondly, the key position of organizational members who interact with customers provides opportunities to share information internally about customer needs and possible improvements [20]. This is referred to as internal influence which refers to individual initiative in communications to the organization and co-workers to improve service delivery by the organization, co-workers, and oneself [21]. Thirdly, customer satisfaction is largely dependent on behavior of the front-line employee who interacts with the customer. Service delivery therefore includes serving customers in a flexible, courteous, conscientious, and responsive manner [22].

### Social exchange theory

Physicians interact with their organization on a daily basis. Social exchange theory has been widely used to increase our understanding of the individual–organization relationship and is considered one of the most influential theories for understanding organizational behaviour [14]. Central to the theory is the norm of reciprocity. This refers to the tendency of organization members to reciprocate beneficial treatment they receive with positive work-related behaviour [23, 24]. Moreover, previous studies have described how individuals seek to enter and maintain a fair and balanced exchange relationship with the organization at which they work [25]. Two types of benefits may influence the exchange: extrinsic (e.g. financial resources) and intrinsic (e.g. gratitude) benefits [26].

Previous research has generally relied on several distinct constructs rooted in social exchange theory to explain organizationally desirable work attitudes and behaviours [27]. In this study, we focus on two central concepts that refer to self-employed physicians—namely noneconomic exchange and economic exchange [9]. To study the former, we draw on the concept of perceived organizational support (POS), which can be described as the global beliefs concerning the extent to which the organization values the employee’s contributions and well-being [11]. To study the latter, we apply the concept of distributive justice (DJ) to measure physicians’ perceptions of their contractual, financial relationship with the hospital. This refers to the perceived fairness of the rewards that an employee receives from the organization [12].

We excluded interactional and procedural justice because these two types of organizational justice do not strictly refer to the contractual relationship between physician and hospital. In this study, we focus specifically on economic exchange by applying the concept of distributive justice to the financial contract.

### Social identification theory

While many empirical studies have found evidence in support of the norm of reciprocity in a wide variety of organizational attitudes and behaviour [16], it has been recently argued that social exchange is more complex than originally conceptualized and personality characteristics may influence the reciprocity dynamic [27]. More precisely, social identification seems to have powerful effects on how physicians read organizational actions [16]. Social identification is the perception of oneness with, or belonging to, a group [28]. Individuals define themselves in terms of their group membership and ascribe themselves characteristics typical of the group [29]. Social identification thus influences how people define themselves by group membership. Therefore social identification impacts how individuals interpret and react to organizational actions and thereby impacts the relationship between the exchange relationship and individuals’ organizational attitudes and behaviour [16]. Identification with a group leads people to see other group members as being relationally close to themselves and to view other group members as “like them” and “on their side”. Hospital administrators are responsible for mediating physicians’ social exchange with their organization. Thus, the perceived relational distance from administrators could theoretically influence physicians’ interpretation of physician–hospital exchanges [15].

An individual can identify with multiple groups and have multiple job-related identities—for instance, physicians can identify with both their organization and with their profession.

Following Hekman and colleagues [15], we propose that physicians’ level of group identification affects their reciprocity behaviour with the hospital by influencing their perceived relationship with hospital administrators. Physician–hospital exchange takes place largely through hospital administrators, and therefore, these relationships shape hospital–physician relationships.

Organizational identification refers to the extent to which the physician defines himself or herself in terms of the organization and leads to the presumption of a common in-group perspective [30]. Since organizational members who identify to a greater degree with the organization may be more receptive to incorporating organizational interests and perspectives into their own outlook, organizational identification could have a beneficial effect to the organization and may help to ensure that staff work in the interest of the organization [30]. More precisely, it has been shown that an employee who has a high level of organizational identification is more likely to perform extra-role behaviour [29]. We therefore propose that the identification of physicians with the hospital affects their response to perceptions of physician–hospital exchange by altering their perceived relationship with their organization.

Professional identification denotes the degree to which physicians identify themselves with their profession [31]. Identification with a group leads people to view non-group members, especially members of rival groups, as being different and unsupportive of their interests [32]. Because administrators are seen as emphasizing organizational concerns over professional needs and the goals and values of the organization (the hospital) and profession (the physician) often conflict, they tend to be rival groups to professionals [33]. Moreover, hospital managers and physicians represent different “tribes”, each with its language, values, and culture [34]. We therefore maintain that professional identification alters physicians’ responses to physician–hospital exchange in a manner opposite to that of organizational identification and thus inhibits the reciprocity dynamic.

## Methods

### Purpose and study framework

**Figure 1:**
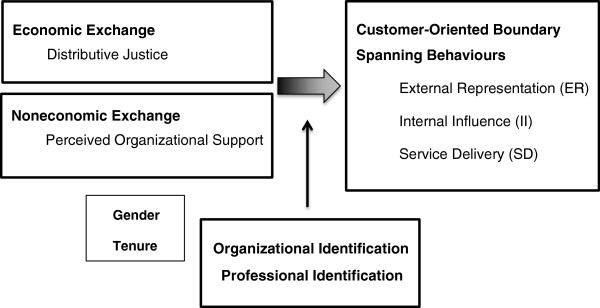
**Study framework.**

The purpose of the study was to examine the effect of self-employed physicians’ perceptions of economic exchange (distributive justice) and noneconomic exchange (perceived organizational support) with the hospital they practice at on their customer-oriented boundary-spanning behaviours (COBSBs). More precisely, we study the external representation (ER), internal influence (II), and service delivery (SD). In addition, we study the moderating effects of physicians’ professional and organizational identification on the relationships between perceived organizational support, distributive justice, and COBSBs (ER, II, and SD). The conceptual framework is based on social exchange theory. Figure 1 provides an overview of the study framework.

Research question 1: Is economic exchange (distributive justice) positively related to physicians’ COBSBs (external representation, internal influence, and service delivery)?

Research question 2: Is noneconomic exchange (perceived organizational support) positively related to physicians’ COBSBs (external representation, internal influence, and service delivery)?

Research question 3: Does organizational identification positively moderate the positive influence distributive justice and perceived organizational support on physicians’ COBSBs (external representation, internal influence, and service delivery)?

Research question 4: Does professional identification positively moderate the positive influence of distributive justice and perceived organizational support on physicians’ COBSBs (external representation, internal influence, and service delivery)?

#### Study design

Data were collected from a survey of self-employed physicians practicing at a convenience sample of six hospitals in Flanders (Belgium). The physicians were invited (and two times reminded) by their CMO to participate in the online survey. The invitation included a letter explaining that this study of Ghent University was supported by the Flemish association of head physicians, the medical board, and executive team of the hospital in which they practice. A concise explanation of the study aim was also included. Out of the 761 physicians from six hospitals in Flanders who were invited to participate, 180 physicians completed the online survey (initial response rate = 27%). After checking the results for missing values, the final sample consisted of 130 physicians. Participation in the study was voluntary and anonymous. The medical ethics committee of the University Hospital of Ghent approved the study.

#### Setting

Belgian physician-specialists practice prevailingly as self-employed professionals. From a financial point of view, physicians have therefore a distinct revenue stream. The hospital is reimbursed for the operating expenses (non-medical activity) by a hospital budget. This budget covers the hotel costs, cost of nursing, etc. The physician is entitled a medical fee for the medical activities, mainly reimbursed by fee for service. However, notwithstanding physicians operate as self-employed practitioners, they need the organizational support that enables them to practice medicine. To cover these costs, a negotiation takes place to determine the share of fees that should be transferred to the hospital (a contract governing the financial relationship).

#### Measures

The survey was collated from previously published instruments, which have demonstrated sound psychometric properties in past research. All question items were translated to Dutch and then back translated in order to ensure that the meaning had been retained—for which three independent translators were used. We used a five-point Likert-type scale (1 = strongly disagree; 2 = disagree; 3 = neither agree nor disagree; 4 = agree; and 5 = strongly agree). Following previous research, the items were aggregated to create a scale score.

Physicians’ perceptions of distributive justice were measured by the four-item scale developed and validated by [35]. The items explicitly addressed the financial, economic relationship. Two sample items are as follows: “Does your financial agreement with the hospital reflects the effort you have put into your work?” and “Is your contract appropriate for the work you have completed?” The internal consistency of the instrument was sufficiently high (Cronbach’s *α* = 0.93).

Physicians’ perceived organizational support was assessed using the eight-item scale of Eisenberger et al. [11] (1986). Cronbach’s *α* for this was satisfactory (0.94). Two sample items are as follows: “Help is available from my organization when I have a problem” and “My organization is willing to help me, if I need a special favour”.

We measured physicians’ customer-oriented behaviours using a six-item shorted version of the scale of Bettencourt and Brown [8]. We used a short version in order to limit the length of our questionnaire. For each type of COBSB, we used two items. Sample items are as follows: “I encourage friends and family to come to this hospital for its products and services” (external representation); “I encourage other coworkers to contribute ideas and suggestions to improve services” (internal influence); and “I take time to understand patients’ needs on an individual basis” (service delivery). The internal reliability for the three scales was acceptable (respectively, *α* = 0.81, *α* = 0.94, and *α* = 0.80). A confirmatory factor analysis (principal component analysis with oblique rotation) extracted three factors with an eigenvalue greater than 1, which corresponded with the three forms of customer-oriented behaviour. A total of 88.3% of variance was explained by the three factors.

The extent to which self-employed physicians identified with the hospital was measured using the five-item scale of Mael and Ahforth [29]. A sample item is as follows: “When someone criticizes the hospital, it feels like a personal insult”. The internal consistency was acceptable (Cronbach’s *α* = 0.83). Following Hekman et al. [15], the extent to which physicians identified with the medical profession was measured with the same basic items and rating scale used to measure OI, but with all references to the hospital changed to “medicine” or synonyms. A sample item is as follows: “When I talk about physicians, I usually say ‘we’ instead of ‘they’”. Cronbach’s *α* was acceptable (*α* = 0.80).

#### Control variables

A demographic questionnaire was included to obtain descriptive information. Gender, age, tenure, and profession (surgery or internal medicine) were included to rule out potential alternative explanations for our findings. Previous research has suggested that these variables are important to social exchange [15].

### Analysis

The Statistical Package for the Social Sciences (SPSS), version 22 for Windows, was used to conduct descriptive and statistical analyses. Correlation analyses were performed to assess possible multicollinearity between the control variables. The age and tenure variables correlated highly (*r* = 0.843), and so age was not used as a control variable. In addition, since profession (surgery or internal medicine) did not correlate with the dependent, independent, and moderating variables, this control was not included. Descriptive statistics were used to describe the sample and study variables. Confirmatory factor analyses confirmed the distinctiveness of the measures used in this study. Pearson correlation analysis was used to test whether the variables were related. To test our research questions and analyses, the data underwent hierarchical regression analysis. To avoid multicollinearity, the independent variables were centred [36]. The first step of the analysis involved entering the control variables, gender, and organizational tenure into the model. In the second step, the centred independent variables were added, and the centred moderating variables were then entered. Having multiplied the centred independent variables by the centred moderators, these two-way interaction terms were entered, while controlling for their main effects and the control variables (gender and organizational tenure). Following Bal et al. [37], we argue that interaction effects may be more difficult to detect (especially in field studies), and so an alpha level of 0.10 was used to estimate interaction effects [38]. To understand the form of these interactions, we plotted the regression lines at 1 standard deviation below and 1 standard deviation above the median.

## Results

The population of the respondents is 38.5% female and 61.5% male. The average age is 46.5 years (SD = 9.14). About half of the sample has practiced for more than 15 years in the hospital. These figures are comparable with the characteristics of the whole medical staff. Non-respondents did not differ from respondents with respect to gender, tenure, age, or specialism.

### Economic and noneconomic exchange and COBSBs

No significant differences in the perceptions of economic exchange (distributive justice), noneconomic exchange (perceived organizational support), and physicians’ COBSBs were present in terms of gender and tenure. As shown in Table 1, the results demonstrated a significant relationship between distributive justice and external representation (*r* = 0.393, *p* < 0.001) and internal influence (*r* = 0.192, *p* < 0.001). Similarly, the results showed a significant relationship between perceived organizational support and internal influence (*r* = 0.458, *p* < 0.001) and external representation (*r* = 0.192, *p* = 0.033). In contrast, no significant relations between distributive justice and perceived organizational support and service delivery were present.

**Table 1:** **Descriptive statistics, correlations, and Cronbach’s alphas (italics)**

Variable	Mean	SD	1	2	3	4	5	6	7	8	9	
1. Gender	—	—	—									
2. Tenure	—	—	−0.187*	—								
3. Distributive justice	3.602	0.851	0.114	0.079	—	*0.929*						
4. Perceived organizational support	3.010	0.883	0.073	−0.029	0.486**	—	*0.938*					
5. Internal influence	3.709	0.931	−0.124	0.045	0.192**	0.189*	—	*0.936*				
6. External representation	3.941	0.872	0.046	0.160	0.393**	0.458**	0.181**	—	*0.831*			
7. Service delivery	4.079	0.719	−0.135	0.024	0.040	−0.060	0.372	0.036	—	*0.797*		
8. Organizational identification	3.559	0.752	−0.058	0.033	0.314	0.424	0.372	0.595	0.191	—	*0.801*	
9. Professional identification	3.288	0.785	−0.097	−0.072	0.138	0.308	0.351	0.406	0.269	0.754	—	*0.827*

*Correlation is significant at the 0.05 level (two-tailed); **correlation is significant at the 0.001 level (two-tailed).

As shown in Table 2, regression analysis showed that distributive justice explained 15.1% of variance in external representation (*β* = 0.381, *p* < 0.001) and 3.5% of variance in internal influence (*β* = 0.208, *p* = 0.020). Perceived organizational support explained 22.0% of variance in external representation (*β* = 0.458, *p* < 0.001) and 3.2% of variance in internal influence (*β* = 0.189, *p* = 0.026).

**Table 2:** **Regression analyses (** ***n*** **= 130 observations)**

	COBS-ER			COBS-II			COBS-SD		
	***β***	Δ ***R*** ^2^	***P*** value	***β***	Δ ***R*** ^2^	***P*** value	***β***	Δ ***R*** ^2^	***P*** value
Main effects									
Constant	3.824	0.151	0.000	4.164	0.035	0.000	4.380	−0.003	0.000
Gender	0.031		0.832	−0.276		0.102	−0.206		0.122
Tenure	0.077		0.115	0.007		0.893	0.047		0.962
Distributive justice (DJ)	0.381		<0.001	0.208		0.020	0.056		0.536
Moderating effects									
DJ × organizational identification	−0.156	0.415	0.056	−0.001	0.123	0.988	—	—	—
DJ × professional identification	−0.122	0.295	0.125	−0.047	0.125	0.595	—	—	—
Constant	3.548	0.220	0.000	3.997	0.032	0.000	4.334	−0.003	0.000
Gender	0.062		0.658	−0.252		0.134	−0.191		0.150
Tenure	0.099		0.034	0.020		0.716	0.004		0.919
Perceived organizational support (POS)	0.458		<0.001	0.189		0.026	−0.051		0.571
Moderating effects									
POS × organizational identification	−0.146	0.428	0.045	0.055	0.119	0.538	—	—	—
POS × professional identification	−0.107	0.311	0.161	−0.007	0.108	0.933	—	—	—

### The moderating effects of identification

**Figure 2:**
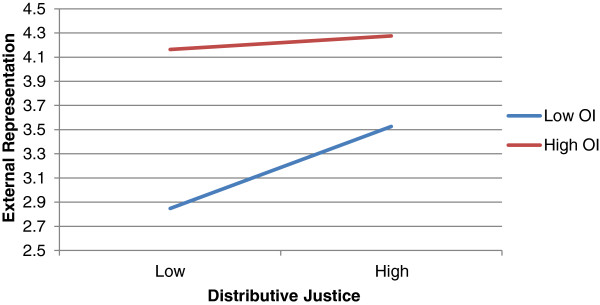
**The moderating effect of OI on the relationship between DJ and COBSB-ER.**

**Figure 3:**
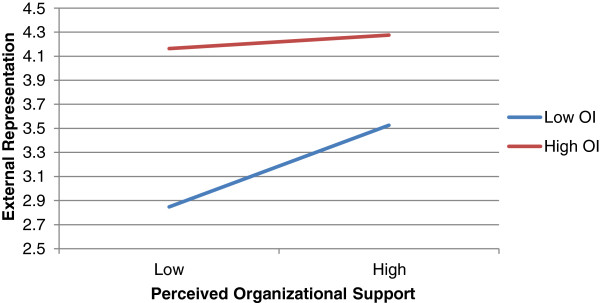
**The moderating effect of OI on the relationship between POS and COBSB-ER.**

As shown in Table 2, organizational identification positively moderated the positive relationship of distributive justice with external representation (*β* = −0.156, *p* = 0.045). Similarly, organizational identification reinforced the positive relationship of perceived organizational support with external representation (*β* = −0.146, *p* = 0.045). In considering the results of internal influence, no moderating effects were present. In addition, professional identification was not found to moderate the studied relationships significantly. The interaction effects are plotted in Figures 2 and 3.

## Discussion

This study is innovative in that it is among the first to study (i) the effects of economic (distributive justice) and noneconomic (perceived organizational support) physician–hospital exchange on self-employed physicians’ customer-oriented behaviours and (ii) the moderating effects of social identification (organizational and professional identification).

The outcomes of this study only partially supported the proposed conceptual model. First, we found that economic and noneconomic exchange has indeed a significant impact on COBSB-external representation. This confirms the spillover effects towards customer-oriented behaviours. However, in considering the results of COBSB-internal influence, it is important to note that, while significant, only a limited percentage of the variance could be explained. A potential explanation for this lower amount of explained variance could be that, in contrast to external representation, internal influence refers to taking initiative actively (which goes beyond communication). It is therefore likely that internal influence is more difficult to realize and influence. In addition, in considering the COBSB-service delivery results, we found no significant relationship. We argue that this can be explained by the fact that service delivery may not be influenced by perceptions of physician–hospital exchanges because physicians—as professionals—are considered the primary advocate of their patient, and thus, COBSB-service delivery should not depend on the physician–hospital exchange.

Second, we found that organizational identification positively moderates the relationship between economic and noneconomic exchange and COBSB-external representation. This was however not the case when COBSB-internal influence is considered. In light of our finding that the amount of explained variance of economic and noneconomic exchange in COBSB-internal influence is relatively low, this is not very surprising. Third, we did not find that professional identification moderates the relationships between the perceptions of exchange and the customer-oriented behaviours of physicians. This is rather surprising in light of our line of reasoning. A possible explanation is that customer-oriented boundary-spanning behaviour refers to a spillover effect between perceptions of organizational treatment by an individual and customer attitudes. Since the level of professional identification (in contrast to organizational identification) does not directly refer to the relationship between individual and organization, this could explain our null result. In addition, we note that our results did not confirm the theoretical argument of rivalry since organizational and professional identification correlated positively. It is therefore unlikely to expect that professional identification alters physicians’ responses to physician–hospital exchange in a manner opposite to that of organizational identification and thus inhibits the reciprocity dynamic.

The main practical implications of this study lay in providing evidence that (i) positive perceptions of physicians of both economic and noneconomic exchange increases physicians’ customer-oriented boundary-spanning behaviours and (ii) organizational identification reinforces this dynamic. With respect to the former (reciprocity), our findings demonstrate that perceived organizational support and distributive justice have an impact on physicians’ customer-oriented behavior directed inside (internal influence) and outside (external representation) the organization. With respect to the latter, we show that organizational identification enhances the positive reciprocity dynamic between physicians and hospital. This stresses the value of this psychological state. Fostering social identification could enhance the reciprocity dynamic, thereby further improving organizational performance. Given the ever-challenging environment hospitals and administrators face, this is an important insight.

Several limitations should be considered when interpreting the results of this study. First, the cross-sectional design of the study does not permit causal interpretations. In addition, we note that the dependent variable external representation correlates highly with organizational identification and perceived organizational support which could result from endogeneity bias. Second, the results should be carefully generalized, as the study focused on a convenience sample of six hospitals in Flanders. Third, no data were collected on physicians who did not respond to the survey. Although the respondents did not significantly differ from the respondents in terms of gender or age, this does not fully rule out representational issues. Additionally, the nonsignificant findings could be related to the limited sample size. However, the results of our study—supported by the theoretical and empirical insights of previous research—are encouraging and suggest that further research is warranted. Moreover, a longitudinal study with a larger sample to examine changes over time would be valuable. Fourth, physicians provided information on both the independent and dependent variables. The use of a common method (a questionnaire) to collect data could lead to bias. However, we reduced the potential for common-method variance by employing measures based on existing scales, proximally separating measures of predictors and the criterion variables and protecting the respondents’ anonymity. Additionally, Harmon’s single-factor tests using factor analysis were conducted. The results showed that none of these factors accounted for the majority of the covariance among the items. We therefore conclude that common-method bias was not a serious threat to our analyses [39].

Finally, it would be valuable to extend this study with other measures of the customer-oriented behaviour of physicians or clinical outcomes. A methodological design involving objective measures of customer-oriented behaviour, or involving patients, peers, and other caregivers, to collect data on physicians’ customer-oriented attitudes would be valuable. In addition, hospital–physician relationships are characterized by an ideologically pluralistic work setting in which professional and administrative roles bump up against each other [40]. Therefore, it would be interesting to differentiate between administrative and professional dimensions of noneconomic physician–hospital exchange [41].

This study provides preliminary evidence that the quality of physicians’ customer-oriented behaviours depends not only on the patient and physician but also on the interaction between the physician and the hospital at which he or she practices. These findings imply that investment in building high-quality relationships with physicians may have an effect on customer-oriented behaviours and thus the patient experience. Future study is needed to further confirm this relationship. Besides quantitative research, a qualitative inquiry or a mixed-method design would be valuable to gain an in-depth understanding of the effects of social identification on physician–hospital reciprocity.

## Conclusion

Our results demonstrate that both perceptions of economic and noneconomic exchange are important to self-employed physicians’ customer-oriented behaviours (external representation and internal influence). However, neither has any impact on customer-oriented service delivery behaviour. Organizational identification reinforces the relationship between economic and noneconomic exchange and external representation behaviour. Professional identification was not identified as a moderator.
